# A Purely Homœopathic Institution

**Published:** 1887-08

**Authors:** 


A PURELY HOMŒOPATHIC INSTITUTION.—It is not often that a dean of a homœopathic college will openly acknowledge that his institution is not homœopathic in its teaching.

Most of these deans have the good sense to keep silent; but of such is not the dean of the "Hahnemann" Medical School, of this city. This worthy dean is reported by the *Record* (Phila., May 30, '87) to "assert positively that the 'Hahnemann' is and always was a purely homœopathic institution."

To show *his* idea of pure Homœopathy the dean added he had "administered Morphia as a palliative, and if he did not continue to do so he would be open to severe criticism."

"Open to severe criticism" from whom? Certainly not from homœopaths, who never need the aid of palliatives. "Open to severe criticism" from his colleagues in the "Hahnemann" College faculty? Hardly, for this same dean also declared that his faculty were unanimous, that "there is no division among them." Hence they could not severely criticise this dean; it is not at all likely they would be allowed to differ from their dean in theory or in practice. He is an autocrat who allows no disobedience among his followers.

The legislators who appropriate public money, and citizens who donate private funds, will know what kind of an institution they assist. Moreover, physicians will also know for a certainty where to send their pupils, that they may learn the homœopathic uses of the hypodermic syringe.

The "*Hahnemannian*" *Monthly* has asserted that there *are* logical reasons for deserting Homœopathy for eclecticism. Perhaps to escape "severe criticism" is one of those reasons.

